# Preventing effect of plant extracts and omega-3 on age-related cognitive decline in male mice

**DOI:** 10.1016/j.bbih.2025.101127

**Published:** 2025-10-23

**Authors:** Marie Martin, Adrien Peltier, Heena Vanmalibhai Lad, Aline Foury, Charlotte Madore-Delpech, Line Pourtau, David Gaudout, Sophie Layé, Véronique Pallet, Anne-Laure Dinel, Corinne Joffre

**Affiliations:** a Université Bordeaux, INRAE, Bordeaux INP, Nutrineuro, UMR 1286, 33076, Bordeaux, France; b Activ’Inside, 12 route de Beroy, ZA Du Grand Cazeau, 33750, Beychac-et-Caillau, France; c NutriBrain Research and Technology Transfer, NutriNeuro, 33076, Bordeaux, France

**Keywords:** Aging, Age-related cognitive decline, Memory, Microbiota, Polyphenols, Saffron, Omega-3

## Abstract

**Background:**

Aging is associated with cognitive decline, accompanied by neuroinflammation, oxidative stress, impaired synaptic plasticity, and gut microbiota dysbiosis. Nutritional interventions, specifically those rich in polyphenols, carotenoids, and omega-3 (om-3) fatty acids, have demonstrated potential benefits in preventing age-related cognitive decline. This study investigates the combined effects of plant extracts (PE) including polyphenols and carotenoids, and fish oil containing om-3 on cognitive function and underlying biological processes in aged mice.

**Objectives:**

To assess the impact of PE and om-3, alone and in combination, on age-related cognitive decline, especially on memory outcomes and to highlight mechanisms involved in these effects.

**Methods:**

17-month-old male C57BL/6 J mice were divided into control and supplemented groups (PE, om-3, and PE + om-3). A group of young mice was used for positive control. Behavioral assessments, including the Elevated Plus Maze (EPM) for anxiety-like behavior, the Object Location Test (OLT) for short-term memory and the Morris Water Maze (MWM) for long-term memory, were conducted. RNA sequencing, fatty acid and oxylipin concentrations analysis and gut microbiota analysis were used to explore molecular changes and microbial diversity.

**Results:**

All supplementations significantly improved short-term memory in the OLT, while only PE and PE + om-3 prevented long-term memory deficits in the MWM. All supplementations modulated gene expression, reducing inflammation, apoptosis or oxidative stress markers in the hippocampus. PE + om-3 further enhanced synaptic plasticity pathways and improved microbiota composition by decreasing harmful bacteria associated with cognitive decline.

**Conclusion:**

Combined PE and om-3 supplementation could provide a complementary approach to combat age-related cognitive decline, highlighting its potential in promoting long-term neuroprotection through modulation of inflammation, oxidative stress and gut microbiota.

## Highlights

• The combination of plant extracts and omega-3 prevent age-related cognitive decline.
• Combined supplementation reduces hippocampal inflammation and oxidative stress.
• The combination upregulates synaptic development signalling pathways in hippocampus.
• The combination beneficially modulates gut microbiota linked to cognitive functions.

## Graphical abstract

**Figure:**
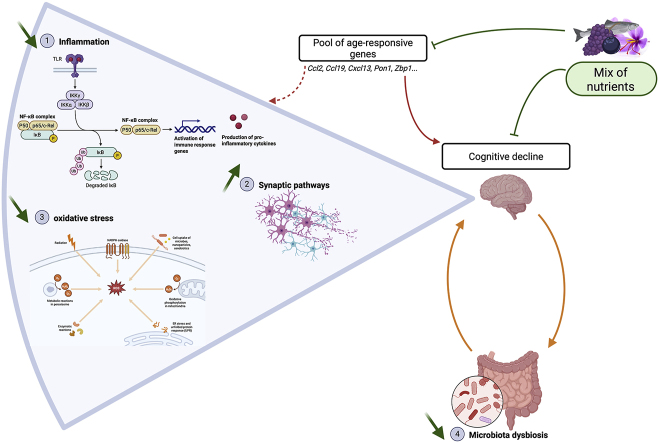

## 1 Introduction

Brain aging is accompanied by a cognitive decline, including memory loss, that concerns 15–20 % of the population aged 65 and over and impairs the quality of life (Ren et al., 2013). Age-related cognitive alterations have been associated with a chronic low-grade inflammation, resulting from an imbalance between pro- and anti-inflammatory cytokine and oxylipin production (Caligiuri et al., 2017; Mooijaart et al., 2013; Ye and Johnson, 2001); an increased oxidative stress, resulting from an imbalance of oxidative reactive oxygen species (ROS) production and antioxidative system activity (Berr et al., 2000; Reddy et al., 1998; Sedelnikova et al., 2004); an altered synaptic plasticity, resulting from a decrease of dendritic spines number and length and reduced neuronal excitability and synaptic transmission (Dickstein et al., 2007; Geinisman et al., 1992; Lu et al., 2024); and a microbiota dysbiosis, resulting from an increase in intestinal permeability and a shift of the microbiota composition (Hopkins et al., 2002; Manderino et al., 2017; Thevaranjan et al., 2017).

Nutrition, an extrinsic factor which can modulate cognitive functions through the modulation of inflammation, synaptic plasticity, oxidative stress and microbiota composition, is a good preventive strategy. Different kinds of nutrients, present in the Mediterranean diet, are of particular interest, based on the efficacy of this diet to improve cognition during aging. Polyphenols and carotenoids present in fruits and vegetables have shown improvement of memory due to their antioxidant and neuroprotective properties and the change in microbiota composition they induce (Banskota et al., 2021; Bensalem et al., 2019; Boskabady and Farkhondeh, 2016; Coultrap et al., 2008; Del Bó et al., 2013; Lacombe et al., 2013; Nanda and Madan, 2021; Tsolaki et al., 2016). Omega-3 polyunsaturated fatty acids (om-3) present in fatty fishes have also beneficial effect on cognitive function through their anti-inflammatory and neuroprotective properties (Dyall et al., 2010; Labrousse et al., 2012; Vedin et al., 2008; Yurko-Mauro et al., 2010). As well, they can also modulate the microbiota composition (Noriega et al., 2016; Saeedi Saravi et al., 2021). A combination of these nutrients could have a positive impact on the prevention of age-related cognitive decline since both types of nutrients target different biological processes that are involved in age-related cognitive decline and since lipids are shown as possible enhancer of polyphenol bioavailability (Azuma et al., 2003; Ortega et al., 2009). We previously show that this combination prevents cognitive alterations, inflammation and oxidative stress and enhances neurogenesis in a model of accelerating aging (Martin et al., 2024). We now aimed at identifying possible mechanisms of action of this combination in physiological aged mice. Moreover, the identification of predictive biomarkers for age-related cognitive decline is necessary for an earlier detection to maximize the chances to prevent these alterations. In this context, we evaluated the influence of supplementations consisting in plant extracts (PE) rich in polyphenols from blueberry and grape, carotenoids and safranal from saffron, and/or in om-3 from fish on short- and long-term memory and we determined the main key biological pathways and markers involved through RNA sequencing and gut microbiota analysis.

## 2 Materials and methods

### 2.1 Animals and treatments

**Fig. 1:**
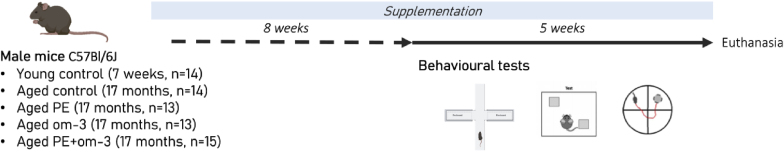
Experimental design.

All experiments were performed with 17-month-old and 7-week-old male C57BL/6 J mice obtained from Janvier Labs (Le Genest-Saint-Isle, France). Mice were maintained under standard housing conditions, on cellulose litter, in a temperature (21 ± 2 °C) and humidity (40 %) controlled environment with a 12 h light/dark cycle (7:00–19:00) and with *ad libitum* access to water and food. Animal husbandry and experimental procedures were done in accordance with the EU Directive 2010/63/EU for animal experiments and were approved by the National Ethical Committee for the Care and Use of Animals (approval ID A27756). A total of 69 animals, separated randomly into 5 groups (n = 13–15/group) were used in this study. Mice were fed with an om-6/om-3 balanced diet supplemented or not with a formulation containing plant extracts (PE) including polyphenols from grape and blueberry extracts (3.69 mg/day, equivalent to 600 mg/day in humans) (Memophenol™ Activ’Inside, Beychac-et-Caillau, France) and carotenoids and safranal from saffron extract (0.1845 mg/day, equivalent to 30 mg/day in humans) (patent pending WO2021209455A1 Activ’Inside, Beychac-et-Caillau, France) and omega-3 (om-3) in triglyceride form (6.15 mg/day of DHA and 1.51 mg/day of EPA, equivalent to 1000 and 245.5 mg/day in humans) or with plant extracts or omega-3 alone (see composition of diets in Table 1). The supplementation began 8 weeks before the beginning of behavioral tests (Fig. 1). At the end of the experiment, mice were euthanized. Brain structure of interest (hippocampus, known to play an important role in learning and memory consolidation (Geinisman et al., 1995)) and caecal content were isolated and stored at −80 °C until analysis.

**Table 1:** Composition of the diets.

	Control	Aged PE	Aged om-3	Aged PE + om-3
**Ingredients (g/kg of diet)**				
Corn starch	460	458.9	460	458.9
Sucrose	230	230	230	230
Cellulose	20	20	20	20
Casein	180	180	180	180
Mineral mix	50	50	50	50
Vitamin mix	10	10	10	10
Fat^a^	50	50	43.5	43.5
**Supplements (g/kg of diet)**				
Memophenol™^b^	0	1.05	0	1.05
Saffron extract^a^	0	0.527	0	0.527
Fish oil (omega-3)	0	0	6.5	6.5

a Rapeseed oil 47 %, sunflower oil 48 %, linseed oil 5 %.

b Total polyphenol ≥75 %: flavonoids (flavan-3-ols, flavonols, anthocyanins) ≥ 43 %, flavan-3-ols monomers ≥20 %, flavan-3-ols oligomers (DP ≤ 4) ≥ 22 %, flavonols (including quercetin and glycosylated derivatives) ≥ 0.15 %, anthocyanins (including malvidin 3-glucoside) ≥ 0.10 %, phenolic acids ≥0.50 %, stilbenes (including resveratrol) ≥ 300 ppm.

a Crocins (mainly trans-4-GG, trans-3-Gg; cis-4-GG, trans-2-G) > 3 %, safranal >0.2 %, picrocrocin derivatives (mainly picrocrocin, HTCC) > 1 %, and kaempferol derivatives (mainly kaempferol-3-sophoroside-7-glucoside, kaempferol-3-sophoroside) > 0.1 %.

### 2.2 Behavioral tests

#### 2.2.1 Elevated Plus Maze

Anxiety-like behavior was evaluated with Elevated Plus Maze test (EPM), as previously described in Dinel et al. (2011). Mice were allowed to freely explore, for 10 min, a plus-shaped acrylic maze with two closed arms (30 × 8 × 15 cm) and two open arms (30 × 8 cm) connected by a central platform (8 × 8 cm), elevated 120 cm above the floor. At the beginning, each mouse was placed in the center of the maze. To analyze time spent in the arms, animals were video tracked using SMART system (Bioseb, Vitrolles, France). A decrease in the percentage of time spent into the open arms is considered an anxiety-like behavior.

#### 2.2.2 Object Location Test

Short-term hippocampus-dependent memory was assessed with an Object Location Test (OLT). This task allows mice to explore objects in a non-threatening and familiar environment. Mice were first acclimatized to the wood open field arena (40 × 40 × 40 cm) the day before the test. The following day, mice were placed for 8 min in the open field with two identical objects and were allowed to explore them for the first part of the test (acquisition). After 1 h of intertrial interval (ITI), mice were placed again for 5 min exploration in the open field but with one of the objects randomly placed in a novel location. To analyze time spent with the objects, animals were video tracked using SMART system (Bioseb, Vitrolles, France) and data were analyzed using The Observer XT software (version 16.0, Noldus, Wageningen, the Netherlands). A recognition index (exploration time of the novel location/total exploration time of both novel and familiar location*100) was calculated to compare the performance of the mice with chance (50 %).

#### 2.2.3 Morris Water Maze

Spatial learning and long-term memory were assessed in the Morris Water Maze test (MWM), as previously detailed in Bensalem et al. (2016). Briefly, mice were habituated to water and swimming for 2 days in a small pool (60 cm diameter), and were required to find a visible platform, with 3 consecutive trials per day and a 60-s cut-off. Following this, visuomotor deficits were evaluated during one day of cued learning in the MWM (150 cm diameter and 50 cm high). Mice had to locate a visible platform marked by a cue, with 6 trials and a 90-s cut-off. For spatial learning, mice were trained over 4 consecutive days to find a submerged platform using distal extra-maze cues, with 6 trials per days and a 90-s cut-off. Mice were placed in the maze from 4 different starting points, randomized daily. Latency and distance to reach the platform, as well as the speed and the swim path of each trial were recorded using Imetronic videotracking system (Pessac, France). 72 h after the last day of training, spatial memory was evaluated with a 60-s probe test in the MWM without the platform. Time spent in each of the four quadrants were recorded using SMART system (Bioseb, Vitrolles, France), with the quadrant where the platform was located during spatial learning referred to as the target quadrant. A recognition index (exploration time of the target quadrant/total exploration time*100) was calculated to compare the performance of the mice with chance (25 %).

### 2.3 RNA sequencing

RNAs were extracted from hippocampi using the Allprep DNA/RNA/Protein Mini (Qiagen, Hilden, Germany) according to manufacturer's protocol. Bulk tissue RNA quality check and RNA-seq was performed by the University of Chicago Genomics Core. RNA concentration and purity were confirmed by Bioanalyzer 2100 (Agilent Technologies). All samples had a RIN score above 9.0. TruSeq stranded cDNA libraries were constructed using Oligo-dT mRNA method. RNA-seq was performed (approximately 50–60 million paired-end reads) using Illumina NovaSeq 6000 at the Chicago Genomics Core facility.

### 2.4 Lipid analysis

Lipid analysis of the hippocampus was performed by the Center des Sciences du Goût et de l’Alimentation (CSGA, INRAE UMR 1324, Dijon, France) as previously described (Labrousse et al., 2012; Lafourcade et al., 2011; Larrieu et al., 2012). After lipid extraction, fatty acids were methylated following the procedures detailed in previous studies (Labrousse et al., 2012; Lafourcade et al., 2011; Larrieu et al., 2012). The resulting fatty acid methyl esters (FAMEs) underwent analysis via gas chromatography using a Hewlett Packard Model 5890 series II gas chromatograph (Palo Alto, CA, USA). This system was equipped with a split/spitless injector, a flame ionization detector (Palo Alto, CA, USA), and a CPSIL-88 column (100 m × 0.25 mm internal diameter; film thickness, 0.20 μm; Varian, Les Ulis, France). Hydrogen was employed as the carrier gas at an inlet pressure of 210 kPa. The process involved maintaining the oven temperature at 60 °C for 5 min, then increasing it to 165 °C at a rate of 15 °C/min, holding for 1 min, and subsequently raising it to 225 °C at 2 °C/min. Finally, the temperature was held at 225 °C for 17 min. The injector and detector temperatures were maintained at 250 °C and 280 °C, respectively. FAMEs were identified by comparison with commercially available standards. The fatty acid composition is expressed as a percentage of the total fatty acids detected.

### 2.5 Oxylipin quantification

Targeted lipidomic was performed at the METATOUL platform (MetaboHUB, INSERM UMR 1048, I2MC, Toulouse, France). The distinct metabolites originating from linoleic acid (LA), arachidonic acid (AA), α-linolenic acid (ALA), DHA, and EPA were isolated from the hippocampus and underwent analysis via mass spectrometry (LC-MS/MS), following the methods outlined by Le Faouder et al. (Le Faouder et al., 2013). Results are expressed as pg/mg of protein.

### 2.6 Microbiota analysis

These analyses were performed at the UMGC Microbiome Services platform (Genomic Center of the University of Minnesota, USA). Genomic DNA was extracted from the fecal content using the QIAamp DNA Stool Mini Kit (Qiagen, Hilden, Germany), which involved a bead-beating step following protocol Q as described by Costea et al. (2017). Subsequently, the concentration of double-stranded DNA (dsDNA) was measured using the NanoPhotometer® GUT MICROBES e2007042-13 Spectrophotometer (Implen, Westlake Village, CA, USA). To analyze the gut microbiota composition, Illumina sequencing of the 16 S rRNA gene was performed. The V3-V4 region of the 16 S rRNA gene was enriched via PCR using primer pairs V3F_Nextera (CCTACGGGAGGCAGCAG) and Meta_V4_806 R (GGACTACHVGGTWTCTAAT). The resulting amplicons were purified, quantified, and sequenced using an Illumina Miseq, generating 2x300-bp sequencing products at the University of Minnesota Genomics Center. During the sequencing run, each base call was assigned a quality score following Illumina's quality scoring methodology, with a mean quality score per sample exceeding 33.8. The sequence reads were automatically converted to FASTQ using a bcl2fastq converter. The obtained 16 S rDNA amplicon sequences underwent analysis using the FROGS pipeline (Escudié et al., 2018). Amplicons were filtered based on size, then clustered into Operational Taxonomic Units (OTUs) using Swarm (with aggregation parameter d = 1+d = 3). Chimeras were eliminated using VSEARCH in combination with innovative chimera cross-validation, and OTUs were retained if they represented more than 0.005 % of the total number of sequences (Bokulich et al., 2013). The OTUs were classified using the reference database Silva 138 16 S, employing a pintail quality of 100 (Quast et al., 2012). The relative abundance of each OTU was calculated after data normalization using a threshold of 33133 reads per sample. Quantitative Polymerase Chain Reaction (qPCR) targeting the 16 S rDNA was used to quantify the abundance of total bacteria and Bifidobacterium spp. PCR amplification was conducted under specific conditions, and detection was achieved using the QuantStudio 3 instrument and software (Applied Biosystems, Foster City, CA, USA), employing the GoTaq qPCR MasterMix Plus for SYBR Assay (Promega). Bovine Serum Albumin (BSA) was added to the samples, and each assay was performed in duplicate within the same run. Standard curves for quantification were constructed using fivefold dilution series from target species genomic DNA preparations (DSMZ, Braunshweig, Germany).

### 2.7 Statistical analyses

Statistical analyses were performed using GraphPad Prism software (v.10.1.0, GraphPad Software, Boston, MA, USA). Young and aged control groups were compared using an unpaired *t*-test or a Mann-Whitney test when data were non-normal. The 4 aged groups were compared using a 1-way ANOVA test followed by Dunnett's post hoc test when appropriate or a Kruskal-Wallis test followed by Dunn's post hoc test when appropriate, when data were non-normal.

A one-sample *t*-test was used to compare the five experimental groups against the expected chance level of 50 % in the OLM and against the chance level of 25 % in Probe test of MWM.

For the microbiota analysis, we utilized the QIIME pipeline (v.1.9.0) to process the sequenced data. Operational Taxonomic Units (OTUs) were selected based on the Greengenes database (v.13.8). Bioinformatics analyses, including evaluations of gut bacterial diversity — specifically alpha and beta diversity — were conducted using the MicrobiomeAnalyst web interface (v.2.0, available at https://www.microbiomeanalyst.ca/, Canada). Additionally, Pearson's correlation analysis was performed on the control groups using R Statistical Software (R Core Team, Vienna, Austria).

For RNAseq analysis, paired-end reads were trimmed using Trimmomatic (Bolger et al., 2014). Trimmed and paired-end reads only were aligned using STAR two pass alignment (Baruzzo et al., 2017; Veeneman et al., 2016) indexed to the GRCm39/mmu 39 soft masked genome to identify splice or novel splice sites and re-indexed to quantify reads. Bioconductor packages in R Statistical Software (R version 4.4.1, R Core Team, 2024; https://www.bioconductor.org, v.3.19) were used for downstream read counts and differentially expressed genes (DEGs) analyses. Read counts were obtained with featureCounts (Rsubread v2.16.1). Low-expressed genes were filtered (less than 10 reads in at least 3 samples). DESeq2 (v.1.44.0) (Love et al., 2014) was used for DEG analysis with GLM matrix weighted on experimental group. Aged control group was compared to young control group to validate the aging model. Mice supplemented with PE, om-3 and PE + om-3 were compared to the aged control group. An FDR (false discovery rate) threshold of 5 % and an absolute log_2_ fold change (LFC) greater than 1 were applied to identify DEGs. Genes with an LFC>1 were considered up-regulated while those with an LFC < −1 were considered down-regulated. A gene set enrichment analysis was performed on all genes using the R package clusterProfiler (v.4.12.0) (Wu et al., 2021). We identified Gene Ontology (GO) biological processes terms that were over-represented in the gene data set (FDR 5 %). Gene annotation was done using the BioTools “ENSEMBL Gene ID Converter” (https://biotools.fr/mouse/ensembl_symbol_converter). The dotplots (clusterProfiler package, v4.12.6) show the most over-represented biological pathways in one group compared to another. "Activated" pathways refer to a positive normalized enrichment score (NES), meaning that genes in these pathways are mostly upregulated while "suppressed" pathways refer to a negative NES. To identify which biological categories were regulated by the different groups, we used the upstream regulators generated through the use of QIAGEN IPA (QIAGEN Inc., https://digitalinsights.qiagen.com/IPA). For each pairwise comparison, genes with an absolute LFC change greater than 1 were retained and analyzed by the software. Upstream regulators were generated based on these genes. Only those with a p-value below the 1e^−4^ threshold were kept for further use. Since way more genes were above the LFC threshold in the aged control against young control comparison, we decided to keep only the first 25 upstream regulators to facilitate the reading of the figure. The genes associated to upstream regulators were categorized into corresponding biological pathways. Graphics were done using the GOplot R package (v.1.0.2) (Walter et al., 2015).

All data are presented as means ± SEM unless indicated otherwise, with statistical significance defined as p ≤ 0.05.

## 3 Results

### 3.1 PE, om-3, and the mix of both supplementations do not prevent age-induced anxiety-like behavior

**Fig. 2:**
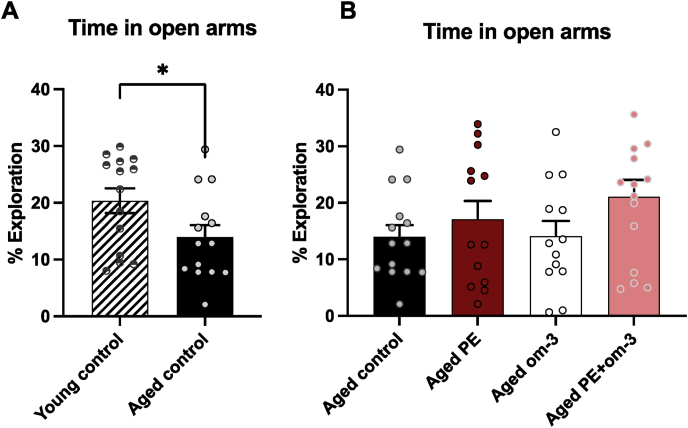
Effect of age and supplementations on anxiety-like behavior in the EPM. Time spent in the open arms. (A) Young control versus aged control (*p < 0.05, unpaired *t*-test, n = 14/group). (B) Non-supplemented versus supplemented aged mice (one-way ANOVA, n = 13–15/group).

As expected, mice in all groups spent more time in closed arms than in novels arms (p < 0.0001 for all groups) (data not shown). As previously described (Chataigner et al., 2021b), aged mice presented anxiety-like behaviors since aged control spent less time in the anxiogenic open arms in the EPM compared to young control mice (t = 2, 112, p < 0,05) (Fig. 2A). Supplementations in PE, om-3 or PE + om-3 did not prevent the age-induced anxiety-like disorders as time in open arms were not significantly different in aged animals (Fig. 2B).

### 3.2 PE, om-3, and the mix of both supplementations prevent age-induced short-term memory deficits

**Fig. 3:**
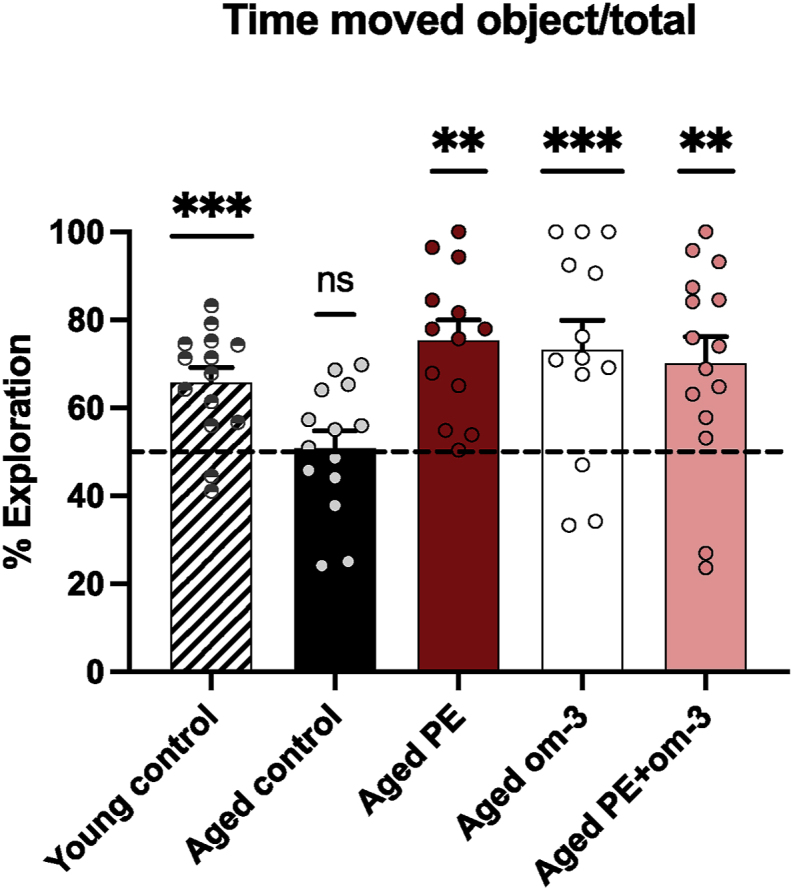
Effect of age and supplementations on short-term memory. Recognition index of the moved object in the OLM after 1 h of ITI. The dotted line corresponds to chance level (50 %) (**p < 0.01, ***p < 0.001 vs chance level by one sample *t*-test, n = 13–15/group).

The short-term memory was evaluated with the Object Location Memory test. The recognition index of the young control group was significantly different from the chance level (50 %), indicating that young control mice correctly discriminated the moved object and did not present memory deficits (p < 0.001) (Fig. 3). As expected, aged control mice did not discriminate the moved object, but mice supplemented with PE, om-3, or PE + om-3 recognized it (PE p < 0.001, om-3 p = 0.004, PE + om-3 p = 0.004). These results suggested that all the supplementations prevented the short-term memory deficits induced by aging.

### 3.3 PE and the mix of PE and om-3 supplementations prevent age-induced long-term memory deficits

**Fig. 4:**
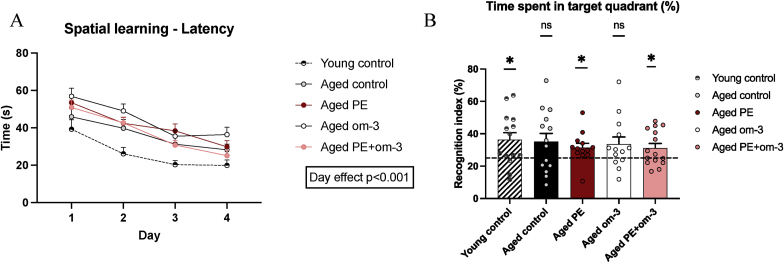
Effect of age and supplementations on spatial learning and long-term memory. (A) Latency to reach the platform during the four days of learning. (B) Recognition index of the target quadrant in the MWM during the probe test. The dotted line corresponds to chance level (25 %) (*p < 0.05 vs chance level by one sample *t*-test, n = 13–15/group).

The effect of the supplementations on spatial learning and long-term memory was evaluated with the Morris Water Maze test. Over the 4 days of learning, all mice significantly spent less time to reach the platform (day effect F_(3,192)_ = 45.33, p < 0.001) indicating that all groups learnt the platform location (Fig. 4A). The recognition index of the young control group was significantly different from the chance level (25 %), indicating that young control mice correctly discriminated the target quadrant during the probe test and did not present memory deficits (p = 0.022) (Fig. 4B). As expected, aged control mice did not discriminate the target quadrant, but mice supplemented with PE, or PE + om-3 recognized it (PE p = 0.029, PE + om-3 p = 0.043). Mice supplemented with om-3 did not discriminate the target quadrant. These results suggested that PE and PE + om-3 supplementations prevented the long-term memory deficits induced by aging.

### 3.4 Om-3 and the mix of PE and om-3 supplementations modulated the n-6/n-3 ratio in the hippocampus

We measured the impact of aging and supplementations on fatty acid composition in the hippocampus (data not shown). Age significantly increased the n-6/n-3 ratio (p = 0.045). Om-3 and PE + om-3 supplementations significantly decreased the n-6/n-3 ratio as compared to the aged control group (F_(3,50)_ = 19.70, p < 0.001) (p < 0.001 and p = 0.007, respectively). There was no significant effect of age and supplementations on total n-6 and n-3 (data not shown).

### 3.5 PE and om-3 supplementations impacted the most oxylipin levels in the hippocampus

**Fig. 5:**
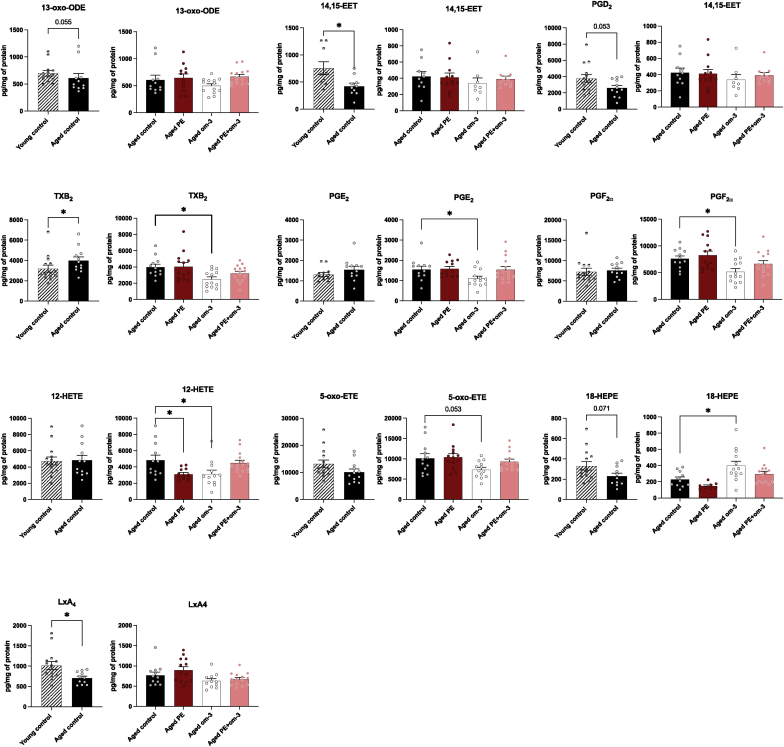
Effect of age and supplementations on oxylipins concentration in hippocampus. (*p < 0.05 by *t*-test vs young control or 1-WAY ANOVA vs aged control, n = 13–15/group).

We evaluated the impact of the supplementations on the levels of oxylipins derived from n-6 and n-3 PUFAs in the hippocampus (Fig. 5). Many oxylipins derived from AA, LA, EPA and DHA were detected. Age significantly decreased the level of protective oxylipins such as 14,15-EET involved in arterial pressure regulation (p = 0.020), PGD_2_ enhancing sleep (p = 0.053), LxA_4_ (p = 0.018) and 13-oxo-ODE (p = 0.055) as anti-inflammatory bioactive molecules derived from AA and LA, respectively, and increased the level of TxB_2_, an inactive product of TxA_2_ that have deleterious vasoconstrictor effect (p = 0.046) derived from AA. The supplementations had beneficial impact on the levels of oxylipins (12-HETE: (H_(3)_ = 14.730, p = 0.002), 5-oxo-ETE: (F_(3,48)_ = 2.967, p = 0.041), PGD_2_: (H_(3)_ = 11.920, p = 0.008), PGE_2_: (H_(3)_ = 10.000, p = 0.019), PGF_2α_: (F_(3,49)_ = 4.569, p = 0.007), TxB_2_: (F_(3,49)_ = 4.250, p = 0.010), 18-HEPE: (F_(3,42)_ = 6.603, p = 0.001). Indeed, PE supplementation significantly increased PGD_2_ (p = 0.013) and significantly decreased 12-HETE involved in inflammation (p = 0.04) as compared to the aged control group. Om-3 supplementation significantly decreased 12-HETE (p = 0.031), 5-oxo-ETE and PGF_2α_ involved in the development of inflammation (p = 0.053 and p = 0.026, respectively), PGE_2_ derived from AA and considered as a main mediator of inflammation (p = 0.038), TxB_2_ (p = 0.014) and significantly increased 18-HEPE (p = 0.010). PE + om-3 supplementation did not have any significant effect on oxylipin composition.

### 3.6 Age and supplementation modulate gut microbiota composition

**Fig. 6:**
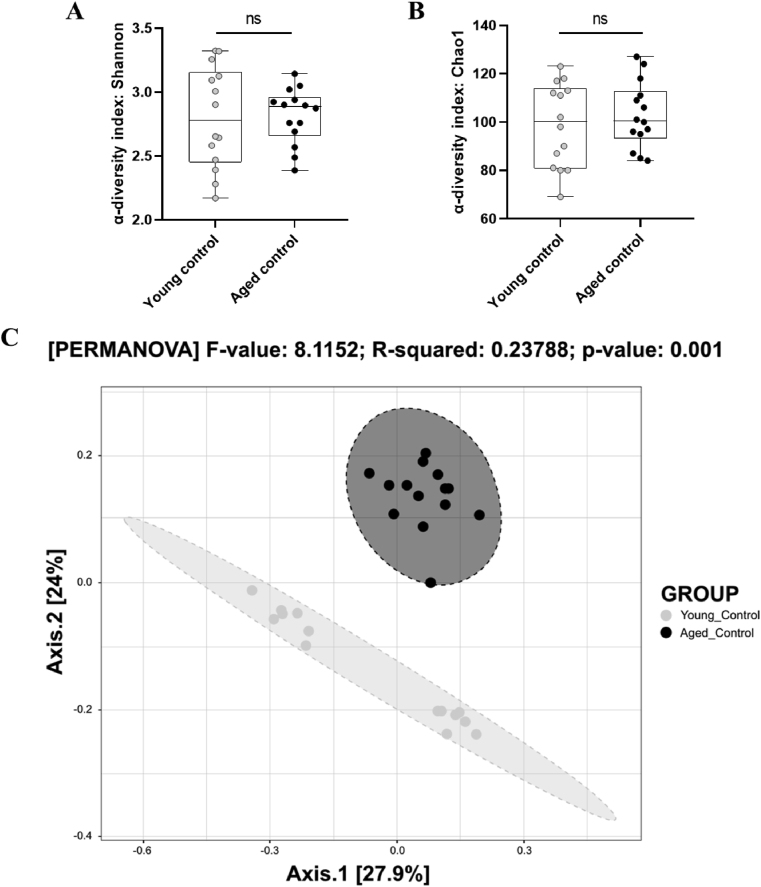
Gut microbial diversity analysis of control groups. (A) Shannon index. (B) Chao1 index. (**p < 0.01 by *t*-test). Data are presented by box and whiskers where whiskers are min to max. (C) PCoA analysis with Bray-Curtis's dissimilarity (n = 14/group).

**Fig. 7:**
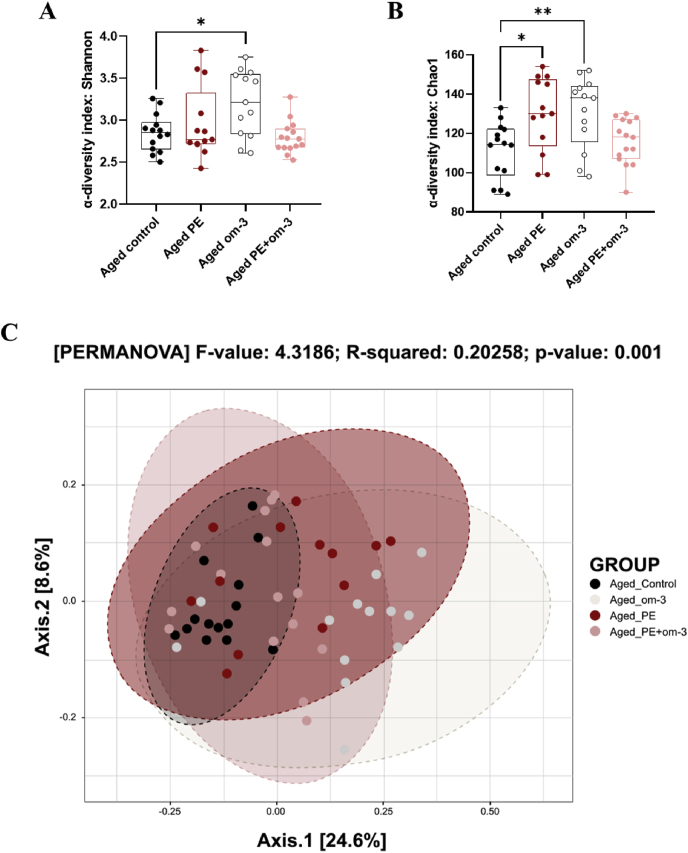
Gut microbial diversity analysis of aged groups, supplemented or not. (A) Shannon index. (B) Chao1 index. (*p < 0.05, **p < 0.01 by 1-WAY ANOVA and Dunnett's post-hoc test). Data are presented by box and whiskers where whiskers are min to max. (C) PCoA analysis with Bray-Curtis's dissimilarity (n = 13–15/group).

Alpha-diversity was assessed using the Chao1 index to measure richness and the Shannon index to evaluate both richness and evenness. Age had no effect on both indices (Fig. 6A–B). The supplementations significantly affected Shannon (F_(3,51)_ = 4.405, p = 0.008) and Chao1 (F_(3,51)_ = 5.183, p = 0.003) indexes (Fig. 7A–B). Specifically, PE (Chao1: p = 0.011) and om-3 (Shannon: p = 0.016, Chao1: p = 0.008) supplementations induced greater diversity than aged control group alone. To further investigate diversity, beta-diversity was also analyzed using PCOA with Bray-Curtis dissimilarity. This revealed differences in microbiota composition between young control and aged control mice (PERMANOVA: p = 0.001) (Fig. 6C), as well as between all aged groups (PERMANOVA: p = 0.001) (Fig. 7C). More precisely, pairwise comparisons between aged groups revealed that all groups significantly differ from each other (p < 0.010 for all).

**Fig. 8:**
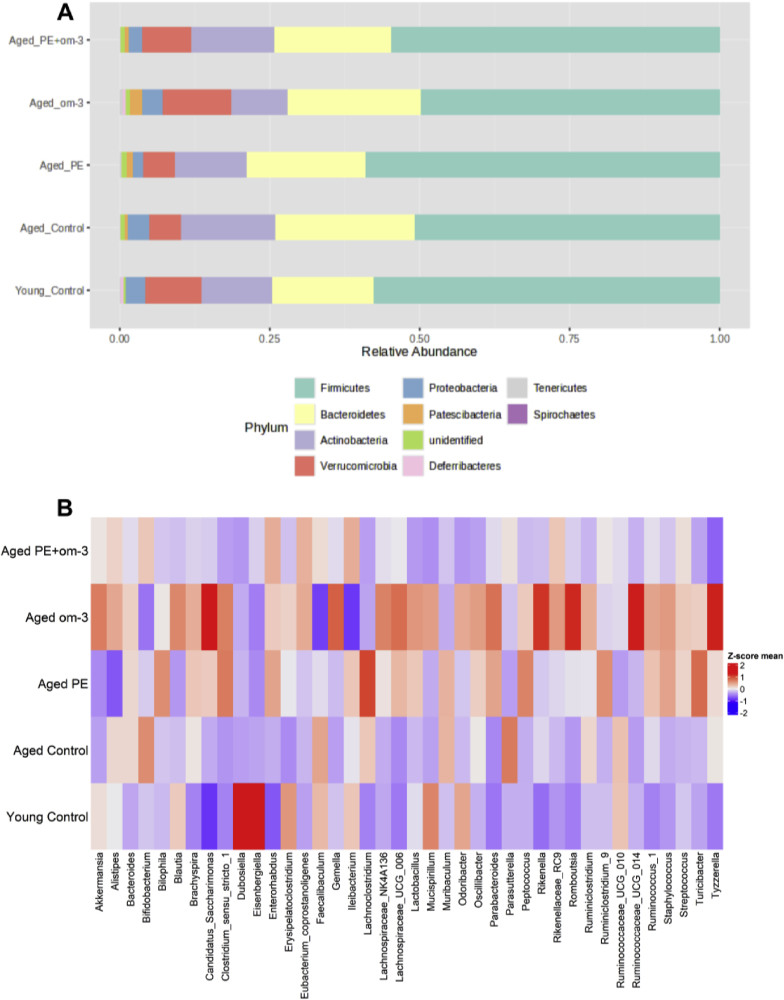
Effect of age and supplementations on gut microbiota composition. (A) Gut microbiota composition at phylum level, (B) Gut microbiota composition at genus level (n = 13–15/group).

Relative abundance of gut microbiota at phylum level was analyzed (Fig. 8A and supplementary data 1). Age significantly affected major phyla. Indeed, it increased the relative abundance of *Bacteroidetes* (p = 0.008), *Actinobacteria* (p = 0.042), and *Patescibacteria* (p < 0.001), and decreased those of *Firmicutes* (p = 0.023) and *Deferribacteres* (p = 0.006). Thus, *Firmicutes*/*Bacteroidetes* ratio was decreased by age (p = 0.001). Supplementation had an effect on the abundance of *Firmicutes* (F_(3,51)_ = 4.079, p = 0.011), *Actinobacteria* (F_(3,51)_ = 3.565, p = 0.020), *Verrucomicrobia* (F_(3,51)_ = 4.226, p = 0.010), *Proteobacteria* (H_(3)_ = 14.870, p = 0.002), *Patescibacteria* (F_(3,51)_ = 9.896, p < 0.001) and *Tenericutes* (H_(3)_ = 7.887, p = 0.048). More precisely, PE supplementation significantly increased *Firmicutes* (p = 0.011) and decreased *Proteobacteria* (p = 0.001) abundances compared to control. Om-3 supplementation significantly increased the abundance of *Verrucomicrobia* (p = 0.013), *Patescibacteria* (p < 0.001) and *Tenericutes* (p = 0.019) and decreased those of *Actinobacteria* (p = 0.007) compared to control. PE + om-3 decreased the abundance of *Proteobacteria* compared to control (p = 0.042).

For clarity, only the most relevant genera are described below, while the full list of statistically significant changes is provided in Supplementary Data 1 (Fig. 8B and supplementary data 1). As highlighted by the heatmap representation, age and supplementation induced modifications of the abundance of several genera, and notably om-3 supplemented mice showed a completely different microbiota composition than the other groups. In fact, 15 genera were significantly affected by age while 18 were significantly affected by supplementation. In particular, aging increased the abundance of *Bacteroides*, *Bifidobacterium*, *Candidatus Saccharimonas*, *Muribaculum*, *Parasutterella*, *Rikenellaceae_RC9*, *Ruminococcus* and *Tyzzerella* while decreasing the abundance of *Dubosiella*, *Eisenbergiella*, *Erysipelatoclostridium*, *GCA_900066575*, *Mucispirillum* and *Ruminococcaceae_UCG_014*. Supplementations further modulated several genera: PE supplementation increased the abundance of *Clostridium _sensu_stricto_1*, *Parabacteroides* and *Turicibacter* and decreased the abundance of *Parasutterella* compared to control. Om-3 strongly reshaped the microbiota with marked increases of the abundance of *Akkermansia*, *Candidatus Saccharimonas*, *Clostridium_sensu_stricto_1*, *Parabacteroides* and *Rikenella*, and a decrease of those of *Bifidobacteriu*, *Muribaculum*, *Parasutterella* and *Turicibacter* compared to control. PE + om-3 decreased the abundance of *Muribaculum*, *Parabacteroides* and *Tyzzerella* compared to control. The full list of genera significantly altered by age or supplementation, and the statistical significance are provided in **Supplementary Data 1**.

### 3.7 Age and supplementation modulate hippocampal gene expression

Differentially expressed genes (p < 0.05 and |log2FC|>1) were identified when comparing the aged control group to the young control group, whereas no significant results were found for comparisons between the aged groups. Therefore, we sought to identify biological pathways that were up- or down-regulated by age or supplementation. To this end, two independent analyses were conducted for each pairwise comparison. First, a gene set enrichment analysis was performed using the GO database. Separately, genes with |log2FC|>1 were selected and analyzed using IPA software to identify upstream regulators, retaining those with a p-value below 1e-4 for further investigation.

**Fig. 9:**
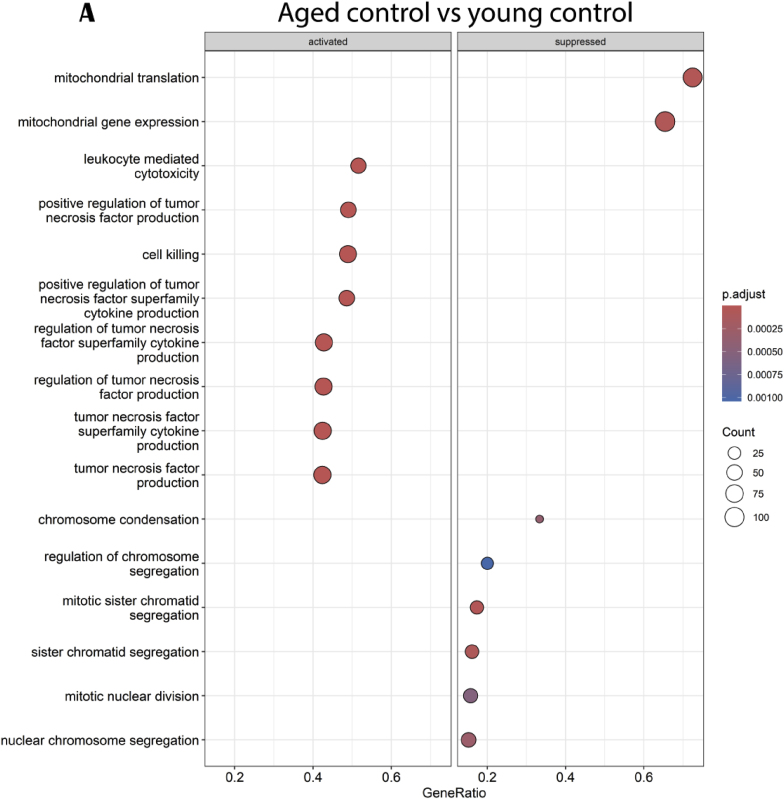

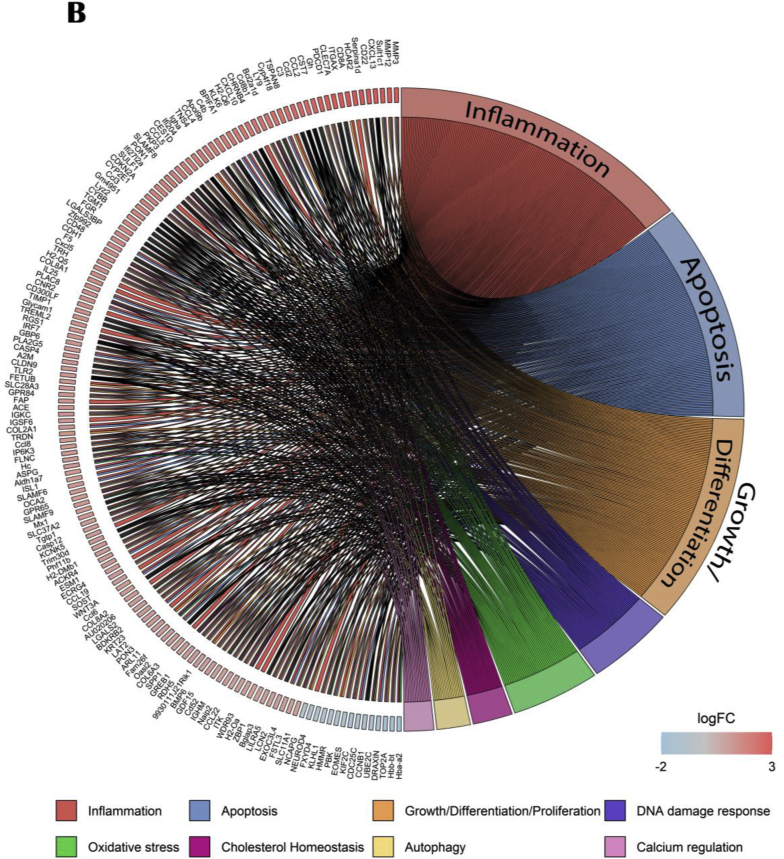
Effects of age on hippocampal genes and pathways expressions. (A) GO enrichment analysis: dotplot of the most over-represented biological pathways activated or suppressed in aged control group compared to young control group, (B) Circos plot of main upstream regulators and their target genes with a |log2FC|>1 in aged control group compared to young control group. Red indicates an upregulation and blue indicates a downregulation (n = 14/group). (For interpretation of the references to colour in this figure legend, the reader is referred to the Web version of this article.)

Compared to young control group, upregulated genes in aged control mice are highly implicated in biological process concerning cytokine production and particularly tumor necrosis factor regulation (Fig. 9A). Down-regulated genes are mostly involved in mitochondrial expression and translation. The Circos plot representation, with 25 upstream regulators, well demonstrated that aged mice presented an upregulation of genes mainly involved in inflammation, apoptosis, cellular growth and differentiation, DNA damage response and oxidative stress (Fig. 9B).

**Fig. 10:**
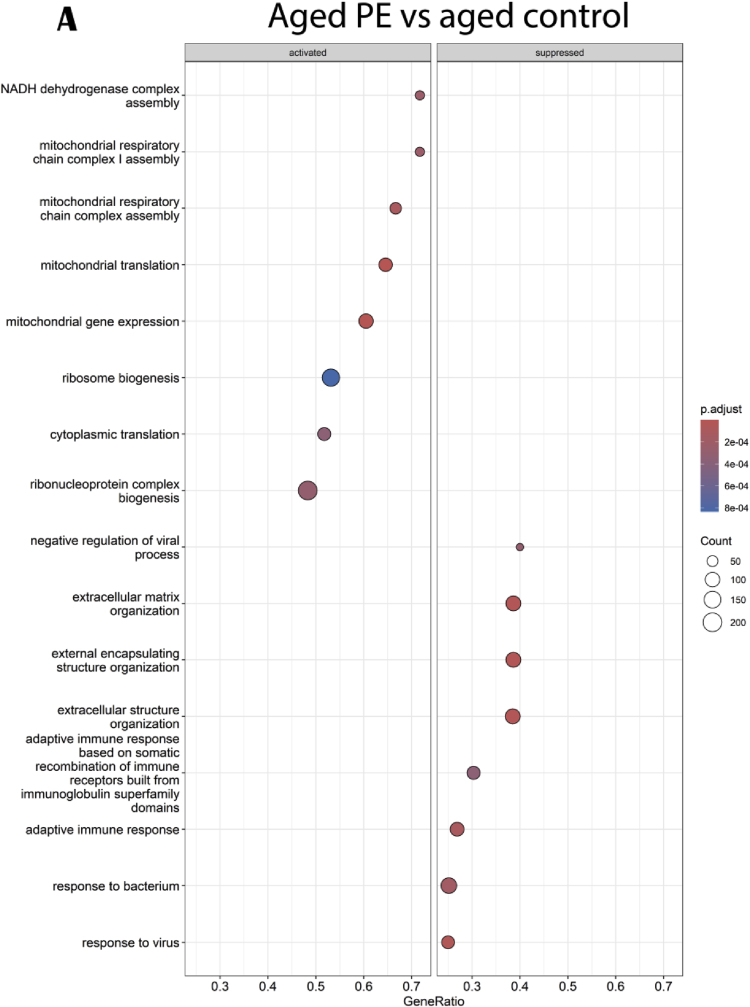

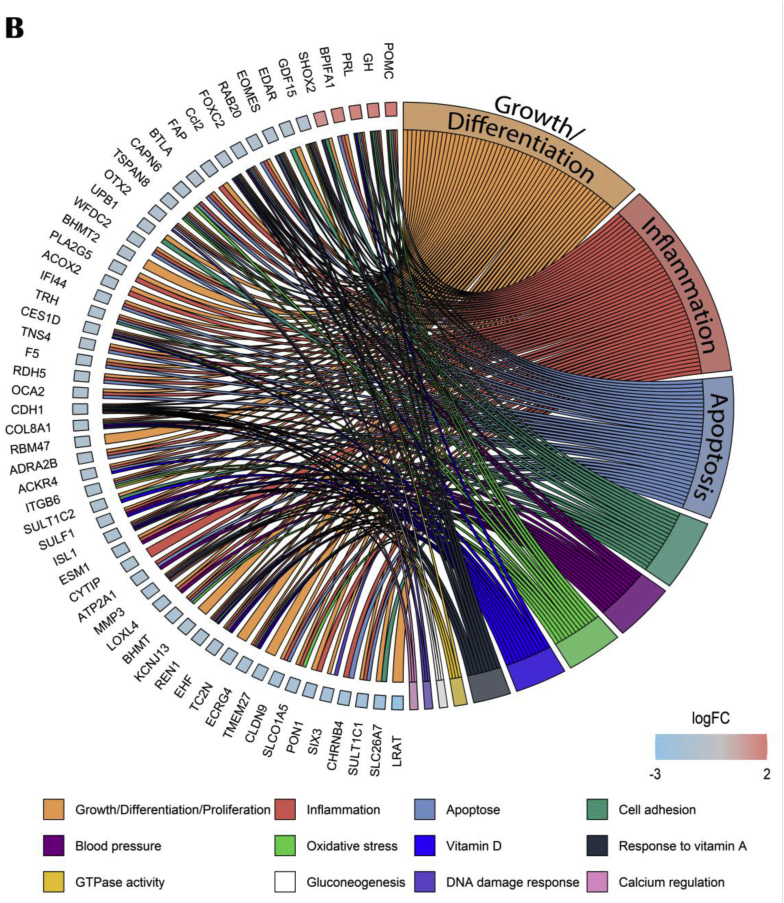
Effects of PE supplementation on hippocampal gene and pathways expressions. (A) GO enrichment analysis: dotplot of the most over-represented biological pathways activated or suppressed in aged PE group compared to aged control group, (B) Circos plot of main upstream regulators and their target genes with a |log2FC|>1 in aged PE group compared to aged control group. Red indicates an upregulation and blue indicates a downregulation (n = 13/group). (For interpretation of the references to colour in this figure legend, the reader is referred to the Web version of this article.)

Interestingly, the aged PE versus aged control comparison showed that activated genes were implicated in mitochondrial and ribosomal associated pathways whereas suppressed genes were implicated in immune response and extracellular organization (Fig. 10A). 35 upstream regulators were identified using IPA software. Circos plot highlighted that aged PE mice presented a down-regulation of genes involved in cellular growth and differentiation, inflammation, apoptosis, cell adhesion, blood pressure and oxidative stress compared to aged control (Fig. 10B).

**Fig. 11:**
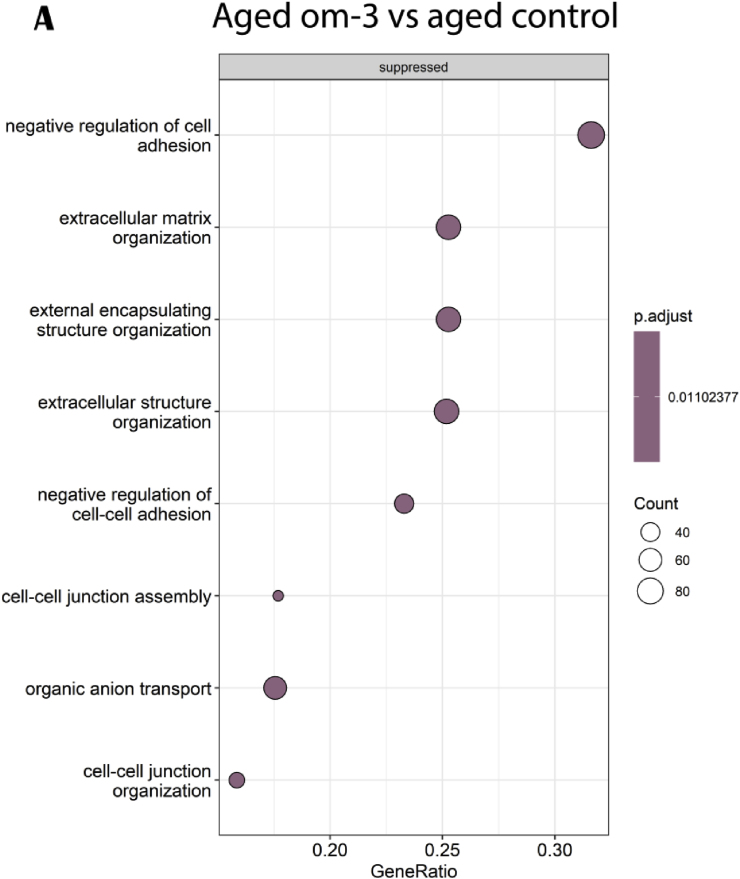

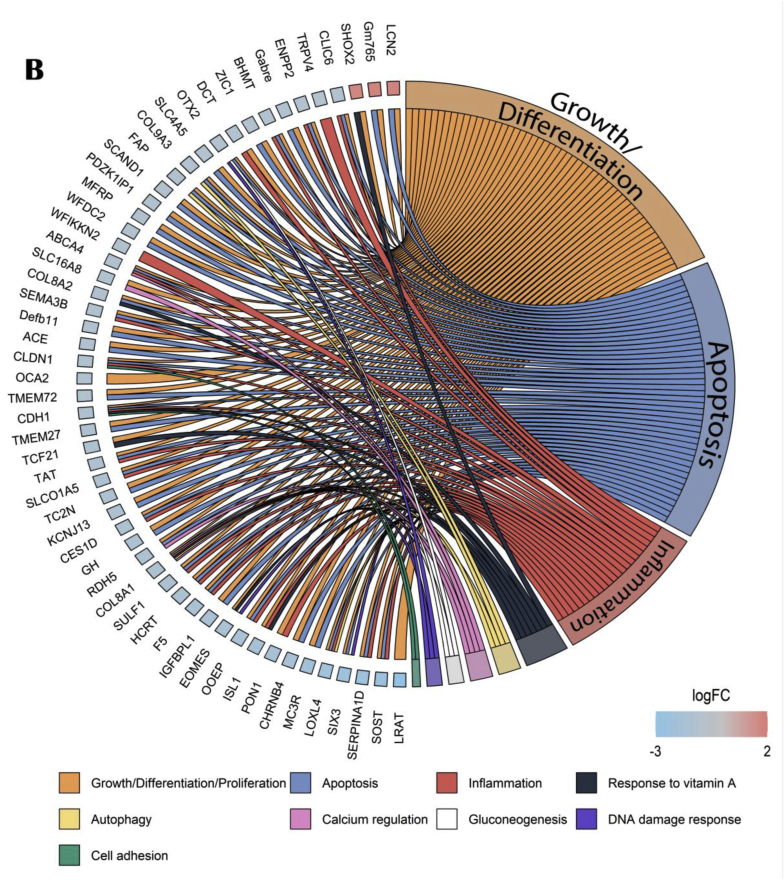
Effects of om-3 supplementation on hippocampal gene and pathways expressions. (A) GO enrichment analysis: dotplot of the most over-represented biological pathways activated or suppressed in aged om-3 group compared to aged control group, (B) Circos plot of main upstream regulators and their target genes with a |log2FC|>1 in aged om-3 group compared to aged control group. Red indicates an upregulation and blue indicates a downregulation (n = 13/group). (For interpretation of the references to colour in this figure legend, the reader is referred to the Web version of this article.)

The aged om-3 versus aged control GO comparison highlighted suppressed genes involved in cells structures as cell adhesion, cell junction and extracellular organization (Fig. 11A). The circos plot, based on 14 upstream regulators using IPA software, confirmed that most of modulated genes were downregulated and implicated in cellular growth and differentiation, apoptosis and inflammation (Fig. 11B).

**Fig. 12:**
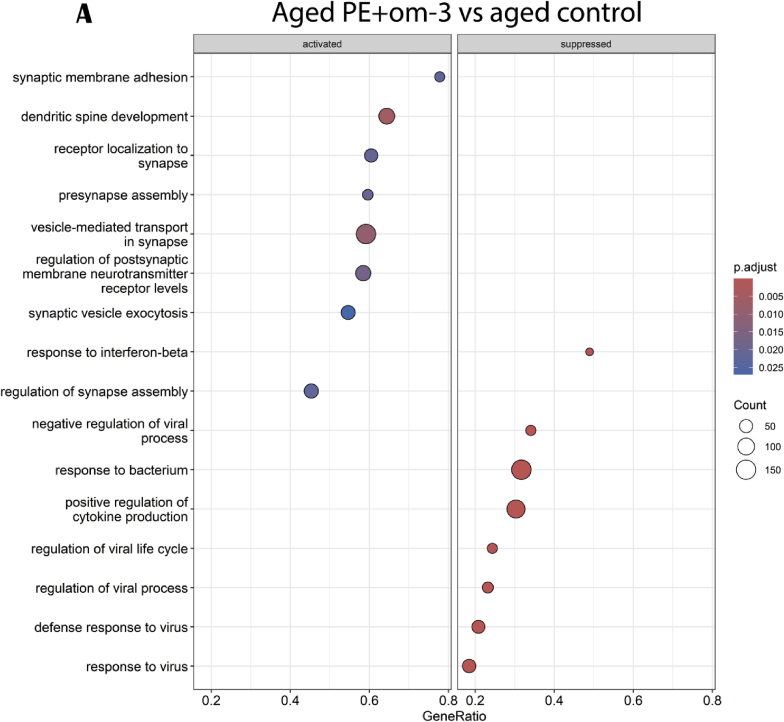

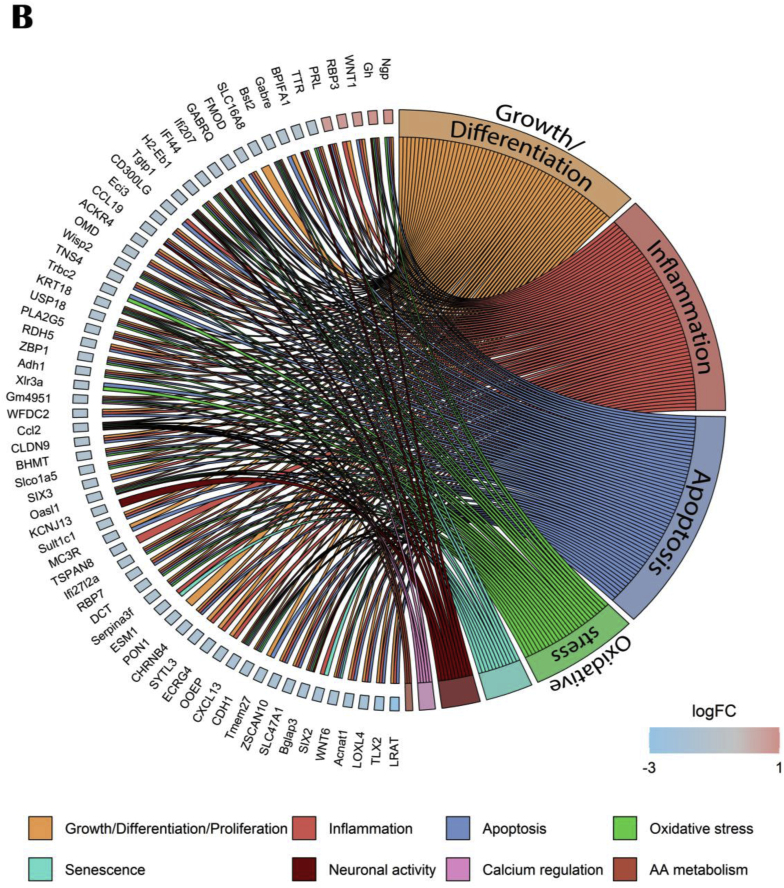
Effects of PE + om-3 supplementation on hippocampal gene and pathways expressions. (A) GO enrichment analysis: dotplot of the most over-represented biological pathways activated or suppressed in aged PE + om-3 group compared to aged control group, (B) Circos plot of main upstream regulators and their target genes with a |log2FC|>1 in aged PE + om-3 group compared to aged control group. Red indicates an upregulation and blue indicates a downregulation (n = 15/group). (For interpretation of the references to colour in this figure legend, the reader is referred to the Web version of this article.)

Finally, PE + om-3 supplementation promoted the activation of genes involved in synaptic pathway (dendritic spine development, exocytosis and transport of synaptic vesicle, presynapse and synapse assembly) and the suppression of genes involved in cytokine production (Fig. 12A). 79 upstream regulators were found using IPA software. Circos plot showed that PE + om-3 supplementation induced a down-regulation of genes involved in cellular growth and differentiation, inflammation, apoptosis, oxidative stress and senescence compared to aged control group (Fig. 12B).

### 3.8 Identification of a pool of age-responsive genes

Based on Circos plot representations, the large-scale gene expression profiling conducted us to identify 19 genes upregulated by aging and downregulated by PE + om-3 supplementation. We then used the raw counts of these age-responsive genes to correlate them with other relevant variables as follows.

**Fig. 13:**
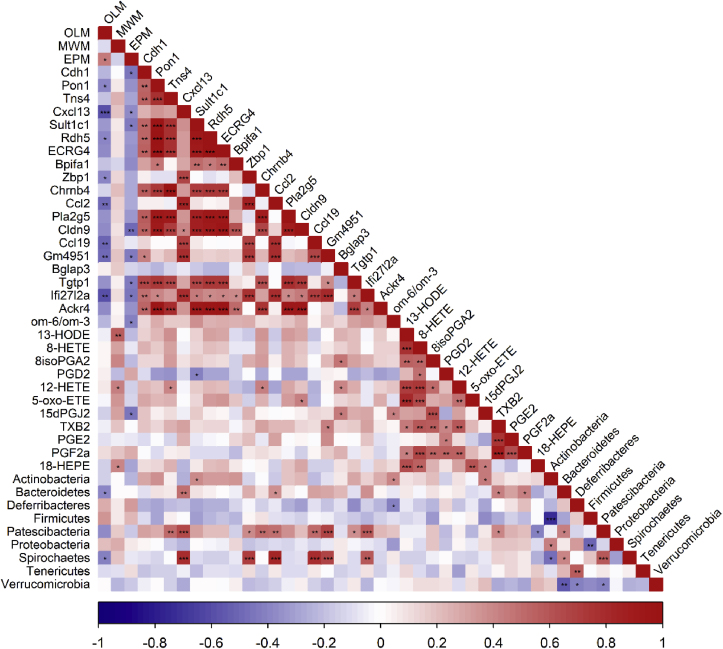
Correlation analysis in control groups. Red indicates higher positive correlation and blue indicates higher negative correlation. (*p < 0.05, **p < 0.01, ***p < 0.001 by *t*-test, n = 14/group). (For interpretation of the references to colour in this figure legend, the reader is referred to the Web version of this article.)

To understand the link between memory, anxiety and the biological markers, we performed a correlation analysis in the population not impacted by supplementation, including both control groups (Fig. 13). We only describe the correlations between measured variables (gene expression, bacteria abundance, oxylipin) and cognitive outcomes. Interestingly, short-term memory performances in OLM were positively correlated with time spent in open arm in EPM, indicating that mice with lower anxiety had better short-term memory. Moreover, OLM performances were negatively correlated with several genes: *Pon1*, *Cxcl13*, *Rdh5*, *Zbp1*, *Ccl2*, *Ccl19*, *Gm4951*, *Ifi27l2a*, as well as *Bacteroidetes* and *Spirochaetes* abundances. EPM performances were also negatively correlated with several genes, including genes in common with OLM performances: *Cdh1*, *Cxcl13*, *Sult1c1*, *Cldn9*, *Gm4951*, *Tgtp1* and *Ifi27l2a*, as well as om-6/om-3 ratio and 15 d-PGJ_2_. Finally, long-term memory performances in MWM were positively correlated with several oxylipins: 13-HODE, 12-HETE, and 18-HEPE.

## 4 Discussion

Aging leads to a decline of cognitive functions associated with biochemical changes and dysbiosis, which can significantly reduce the quality of life. This study demonstrated that age-related cognitive deficits can be prevented through nutritional intervention. Specifically, we observed complementary effects between phytonutrients from plant extracts (Memophenol™ and a patented saffron extract) and omega-3 from fish in preventing age-related alterations. These effects involved modulating pathways related to inflammation including oxylipin profile and om-6/om-3 hippocampal ratio, apoptosis, oxidative stress in the hippocampus and gut microbiota composition.

As previously described, age induced short and long term memory alterations associated with anxiety-like behavior (Chataigner et al., 2021a, Chataigner et al., 2021b). This behavioral pattern was associated with an upregulation of genes involved in inflammation, apoptosis, growth/differentiation/proliferation, DNA damage response and oxidative stress along with a gut microbiota dysbiosis, notably a decrease of *Firmicutes*/*Bacteroidetes* ratio, according to literature (Franceschi et al., 2000; Higami and Shimokawa, 2000; Iakovou and Kourti, 2022; Lombard et al., 2005). Interestingly, we highlighted for the first time a group of age-responsive genes, in hippocampus, negatively correlated with cognitive performance, in particular short time memory (*Pon1*, *Cxcl13*, *Rdh5*, *Zbp1*, *Ccl2*, *Ccl19*, *Gm4951*, *Ifi27l2a*) and positively correlated with anxiety-like disorders (*Cdh1*, *Cxcl13*, *Sult1c1*, *Cldn9*, *Gm4951*, *Tgtp1* and *Ifi27l2a*). Of note, some of these genes have been previously identified as key factors in cognitive alterations and Alzheimer disease (AD). In fact, *Zbp1* is upregulated in AD models *in vivo* in rats and *in vitro* in neurons and its silencing leads to an improvement of memory *in vivo* and a reduction of oxidative stress and inflammation *in vitro* (Guo et al., 2023). *Pon1* is an antioxidant gene involved in the reduction of lipid oxidation that is lower in AD patients but its polymorphism is a risk factor for neurological diseases (Erlich et al., 2006; Paragh et al., 2002; Zhang et al., 2013). In our study, this gene was higher in aged mice but the polymorphism was not known and maybe an overexpression of this gene is detrimental. Moreover, as aging was associated with oxidative stress, we supposed that the expression of *Pon1* increased to fight against oxidative stress. *Cxcl13*, *Ccl2* and *Ccl19* are 3 pro-inflammatory chemokines previously associated with cognitive impairments and that are also upregulated in AD, *Ccl19* being a peripheral biomarker (Chen et al., 2023; Lee et al., 2018; Sorrentino et al., 2021). The other genes listed above are related with inflammation and induced by interferon such as *Gm4951*, involved in lipid oxidation; *Ifi27l2a*, upregulated in microglia in aged brain and even more in aged stroke brain and leading to neuroinflammation through ROS generation; *Tgtp1*, increased in colon inflammation in mice (Breynaert et al., 2013; Kim et al., 2023; Z. Zhang et al., 2022). This suggests that these genes could be relevant biomarkers of cognitive and emotional alterations associated with aging and could be considered as a signature of accelerated aging associated to cognitive decline. Moreover, *Cxcl13* and *Ccl2* were positively correlated with *Bacteroidetes* and *Spirochaetes* abundances that are negatively correlated with short-term memory. Several studies in humans and animals already show a detrimental effect of *Bacteroidetes* on cognition (Nagpal et al., 2020; Saji et al., 2019; Zhang et al., 2021) and *Spirochaetes* have been associated with AD, with the presence of this bacteria in the brain in more than 90 % of cases (Allen, 2016; Miklossy, 2011, 2015). A negative correlation was also observed between om-6/om-3 ratio and performances in EPM test, suggesting a protective effect of om-3 level on anxiety, as already described in adults (Su et al., 2018), but not observed in this study. This may be due to other factors influencing anxiety, such as endocrine system, making the anxiolytic effect of om-3 less visible.

Our results showed that the combined administration of PE and om-3 prevented short- and long-term cognitive deficits in aged mice. While cognitive performance in these tests was comparable to that observed in groups receiving nutrient alone, the real advantages of the PE + om-3 combination became evident at the molecular level. In fact, transcriptomic analysis revealed that the combination of nutrients modulated a wider set of genes, as it modulated 79 upstream regulators (with a p-value under the threshold), 2 and 5 times more than either PE or om-3 alone, respectively. Indeed, the PE + om-3 combination upregulated genes related to synaptic functions pathways like dendritic spine development which is notably induced by long term potentiation (LTP), a form of synaptic plasticity crucial for learning and memory (Morris et al., 1986; Toni et al., 1999). Circos plot analysis revealed that the combination also downregulated genes involved in cellular senescence, characteristic of aging (Zhang et al., 2022). Moreover, the combination also acted on pathways targeted by PE and/or om-3, such as inflammation, growth, apoptosis and oxidative stress. Other studies have already emphasized the positive effects of om-3 on inflammation in aged rodents (Labrousse et al., 2012; Martin et al., 2002; Minogue et al., 2007), the antioxidant properties of polyphenols (Rana et al., 2022) and the anti-inflammatory and anti-apoptosis properties of saffron bioactives (Rajabian et al., 2019). In particular, the combination of nutrients downregulated the expression of the set of age-responsive genes that were negatively correlated with good cognitive ability and performances in OLM and EPM tests. Among these age-responsive genes, it was already demonstrated that pro-inflammatory *Ccl2* is reduced by polyphenols, saffron or omega-3 supplementations, the increase in *Rdh5* in diabetic rats is prevented by resveratrol and *Pon1* is decreased by a high om-3 diet (Abedimanesh et al., 2020; Al-Hussaini and Kilarkaje, 2018; Chataigner et al., 2021a, Chataigner et al., 2021b; DeFuria et al., 2009; Varatharajalu et al., 2010). The regulation of these genes by PE + om-3 supplementation demonstrated the potential impact of this combination on the molecular mechanisms underlying cognitive deterioration. As some of these genes are linked to bacterial abundance, we wondered if the effect of PE + om-3 supplementation could be due to gut microbiota changes as gut microbiota is modified during aging and is communicating bidirectionally with the brain through the gut-brain axis. In fact, the combination of nutrients decreased detrimental bacteria such as *Proteobacteria* and *Tyzzerella* (increased in aged control mice), which are associated with cognitive decline and inflammation, and *Muribaculum* (increased in aged control mice) previously described as a pathobiont, and increased anti-inflammatory *Parabacteroides* (Cui et al., 2022; Shi et al., 2021, 2023; Yang et al., 2023). This extensive pathway regulation could play a role in preventing age-related cognitive decline. Thus, the combination of nutrients offered additional benefits due to its complementary effects on underlying biological processes, modulating additional pathways as nutrients alone.

Concerning nutrients alone, PE downregulated a part of age-responsive genes associated with cognition (*Ccl2*, *Rdh5*, *Pon1*, *Sult1c1*, *Cldn9*, *Cdh1*) as well as om-3 did (*Rdh5*, *Pon1*, *Cdh1*) but only PE supplementation showed a beneficial effect on long-term memory. The lack of effect of om-3 on long term memory, in opposition to what is usually observed (Cutuli et al., 2016; Xia et al., 2023), was associated with a complete shift of gut microbiota composition. Some of these modifications are considered detrimental such as the decrease of *Bifidobacterium*, *Faecalibaculum* and *Ileibacterium*, all described as beneficial or linked to better cognitive functions (Asaoka et al., 2022; Li et al., 2022; Sadovnikova et al., 2021; Zagato et al., 2020); and such as the increase of bacteria associated with lower cognitive performances (*Patescibacteria* phylum, *Rikenella*) (Kim et al., 2021; Zhu et al., 2022) or described as pathogens (*Clostridium_sensu_stricto_1*, *Gemella*, *Ruminococcus*) (García-Lechuz et al., 2002; Rajilić-Stojanović and de Vos, 2014; Wu et al., 2023). However, some of the modifications are beneficial such as the increase of bacteria described as beneficial like *Akkermansia*, *Lachnospiraceae_UGC_006*, *Parabacteroides* and *Romboutsia* (Cani et al., 2022; Cui et al., 2022; Menni et al., 2017; Ye et al., 2021); and the decrease of detrimental bacteria such as the pathobiont *Muribaculum* and *Parasutterella*, associated with chronic inflammation (Chen et al., 2018; Yang et al., 2023). Even if om-3 are often related to an improvement in gut microbiota composition (Pusceddu et al., 2015; Saeedi Saravi et al., 2021), other studies also found mitigated effects, concluding that the relationship between om-3 and microbiota is complex (Balfegó et al., 2016; Noriega et al., 2016; Watson et al., 2018; Yu et al., 2014). PE supplementation had a lighter effect on gut microbiota composition. It increased the abundance of beneficial SCFA producers *Firmicutes* (decreased by age), *Lachnospiraceae_UGC_006* and *Romboutsia*, as well as *Parabacteroides* and *Turicibacter* (Cui et al., 2022; Menni et al., 2017; Sun et al., 2022, 2023; Ye et al., 2021). It also decreased the abundance of *Proteobacteria* (which is higher in mice with cognitive deficits), *Alistipes* (with a trend, negatively associated with a healthful plant-based diet index) and *Parasutterella* (associated with chronic inflammation) (Chen et al., 2018; Shen et al., 2024; Shi et al., 2021). These results confirmed previous studies showing that berry polyphenols and saffron bioactive components can increase the abundance of SCFA-producing bacteria and decrease pathogenic bacteria (Banskota et al., 2021; Han et al., 2020; Osman et al., 2008; Xiao et al., 2020). They validated the hypothesis of a differential effect of plant extracts and omega-3 to prevent age-related cognitive alterations.

Hence, as we previously showed in an accelerated model of aging (Martin et al., 2024), a combination of plant extracts and omega-3 prevented age-related cognitive decline and associated alterations in mice. This study, although successful, has some limitations. First, the combination has to be tested also in female, as it is known that there are differences between sexes in age-related cognitive decline (Benice et al., 2006; Lee et al., 2023) and therefore could be used for dietary interventions in aging populations. Secondly, all the biomarkers were only measured in the hippocampus. It would be interesting to analyze these markers in blood, in order to use them as peripheral biomarkers useful in human studies, predictive for cognitive decline. In fact, a part of the age-responsive genes associated with cognition are already peripheral biomarkers for cognitive alterations such as the chemokines Ccl2 and Ccl19 (Chen et al., 2023; Sanchez-Sanchez et al., 2022).

## 5 Conclusion

Although the combination of plant extract (Memophenol™ and a patented saffron extract) and omega-3 did not produce superior effects on age-related cognitive alterations compared to the nutrients administered individually, it appeared to have a more extensive biological impact, particularly by a larger modulation of gene expression. Additionally, the identification of biomarkers that were correlated with cognitive and anxiety deficits and whose expression was downregulated by the PE + om-3 combination, highlighted crucial underlying mechanisms for protecting against the effects of aging on the brain. This emphasizes the value of a combined nutritional approach to maximize long-term neuroprotection.

## CRediT authorship contribution statement

**Marie Martin:** Writing – original draft, Visualization, Validation, Methodology, Investigation, Formal analysis, Conceptualization. **Adrien Peltier:** Writing – original draft, Visualization, Formal analysis. **Heena Vanmalibhai Lad:** Formal analysis. **Aline Foury:** Formal analysis. **Charlotte Madore-Delpech:** Formal analysis. **Line Pourtau:** Writing – original draft, Validation, Supervision, Project administration, Funding acquisition, Conceptualization. **David Gaudout:** Project administration, Funding acquisition. **Sophie Layé:** Project administration, Funding acquisition. **Véronique Pallet:** Project administration, Funding acquisition. **Anne-Laure Dinel:** Writing – original draft, Validation, Supervision, Project administration, Methodology, Funding acquisition, Conceptualization. **Corinne Joffre:** Writing – original draft, Validation, Supervision, Project administration, Methodology, Funding acquisition, Conceptualization.

## Author disclosures

Activ’Inside funds MM, LP, and DG. AP, HVL, AF, CMD, SL, VP, ALD, and CJ report no disclosures.

## Funding

This work is part of a collaborative project named Silver Brain Food which has been funded by Bpifrance (N° DOS0107224). Other fundings used were Metaprogram DiGit-BIO INRAE DINAMIC and CHESS Region Nouvelle-Aquitaine ExoMarquAge.

## Declaration of competing interest

The authors declare the following financial interests/personal relationships which may be considered as potential competing interests: Marie Martin reports financial support was provided by Activ’Inside SAS. Line Pourtau reports financial support was provided by Activ’Inside SAS. David Gaudout reports financial support was provided by Activ’Inside SAS. If there are other authors, they declare that they have no known competing financial interests or personal relationships that could have appeared to influence the work reported in this paper.

## Data Availability

Data will be made available on request.
