# Supplementary material for: Preventing effect of plant extracts and omega-3 on age-related cognitive decline in male mice
Source: Brain Behav Immun Health. 2025 Oct 23;49:101127. doi: 10.1016/j.bbih.2025.101127 (PMC12596225; doi:10.1016/j.bbih.2025.101127)
Supplement: Multimedia component 1 [file mmc1.pdf]

Supplementary data 1. Effect of age and supplementation on gut microbiota composition at (**A**) phylum and (**B**) genus level. (*p<0.05, **p<0.01, ***p<0.001 by t-test vs Young control or by 1-WAY ANOVA vs Aged Control, n=13-15/group).

A

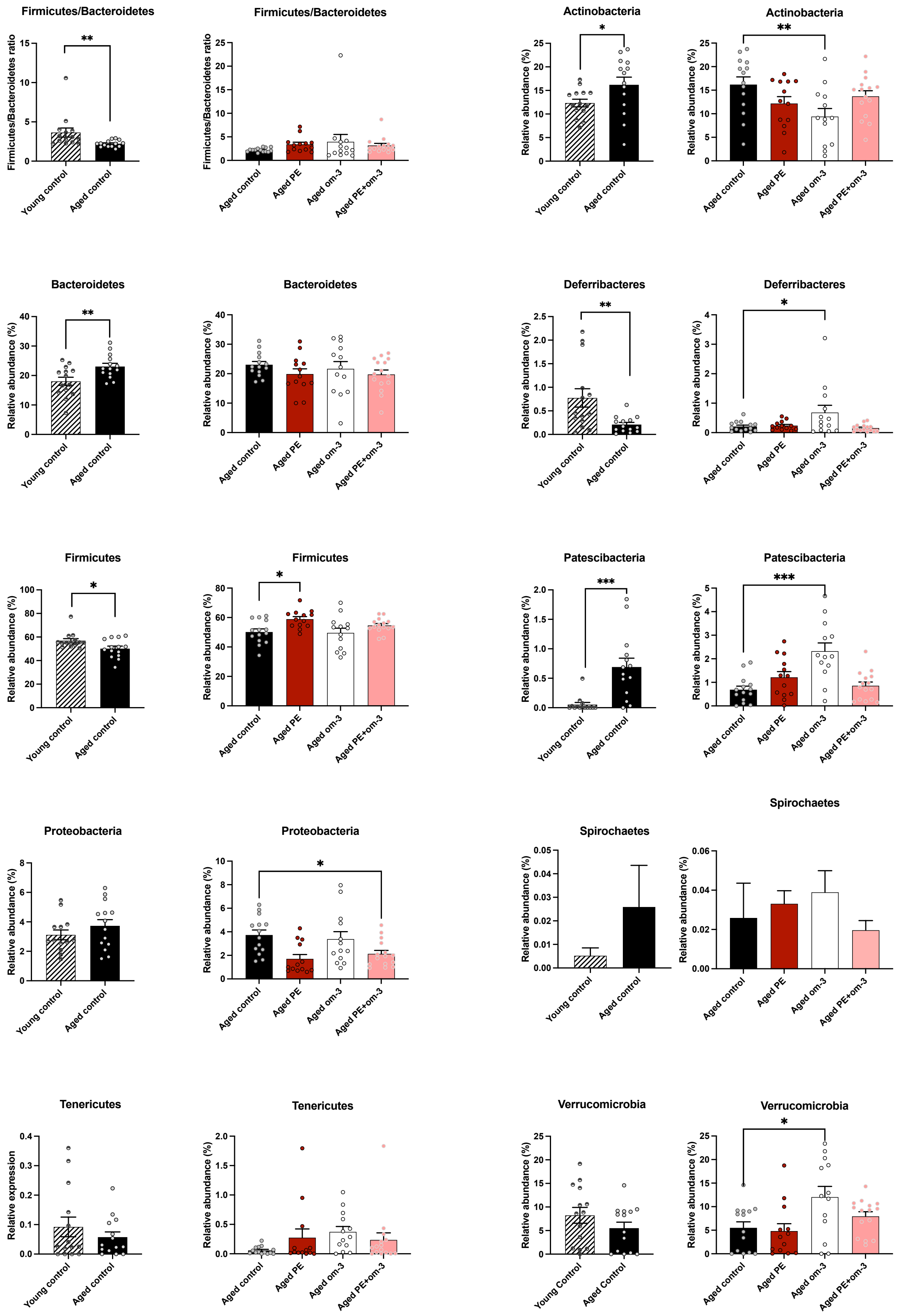

B

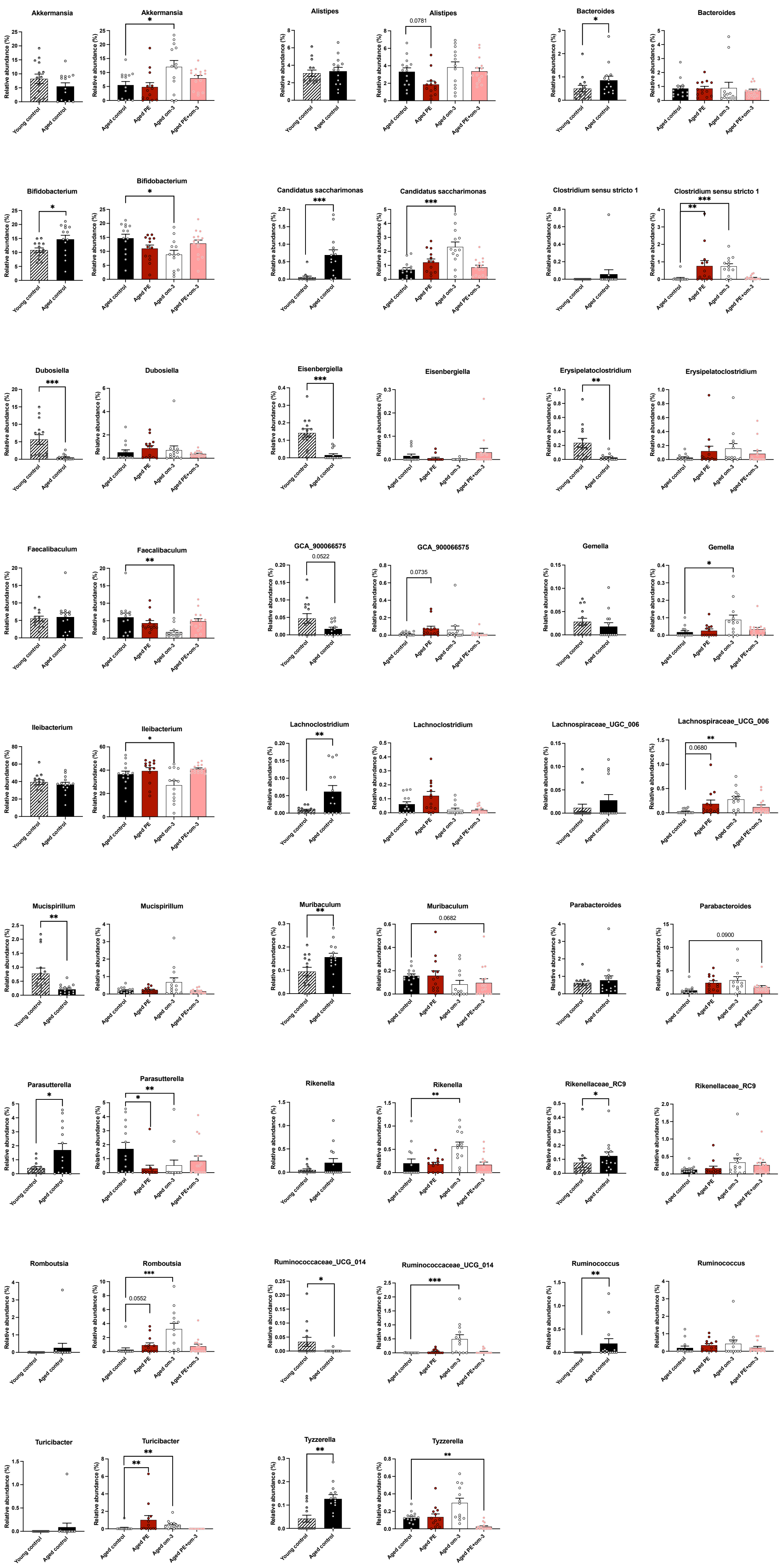

Akkermansia
Akkermansia
Alistipes
Alistipes
0.0781
Bacteroides
Bacteroides
Bifidobacterium
Bifidobacterium
Candidatus saccharimonas
Candidatus saccharimonas
Clostridium sensu stricto 1
Clostridium sensu stricto 1
Dubosiella
Dubosiella
Eisenbergiella
Eisenbergiella
Erysipelatoclostridium
Erysipelatoclostridium
Faecalibaculum
Faecalibaculum
GCA_900066575
0.0522
GCA_900066575
0.0735
Gemella
Gemella
Ileibacterium
Ileibacterium
Lachnoclostridium
Lachnoclostridium
Lachnospiraceae_UGC_006
Lachnospiraceae_UCG_006
0.0680
Mucispirillum
Mucispirillum
Muribaculum
Muribaculum
0.0682
Parabacteroides
Parabacteroides
0.0900
Parasutterella
Parasutterella
Rikenella
Rikenella
Rikenellaceae_RC9
Rikenellaceae_RC9
Romboutsia
Romboutsia
0.0552
Ruminococcaceae_UCG_014
Ruminococcaceae_UCG_014
Ruminococcus
Ruminococcus
Turicibacter
Turicibacter
Tyzzerella
Tyzzerella
Relative abundance (%)
Young control
Aged control
Aged PE
Aged om-3
Aged PE+om-3

Genera description: 15 genera were significantly affected by age while 18 were significantly affected by supplementation. An increase of the abundance of *Bacteroides* ($p=0.0395$), *Bifidobacterium* ($p=0.0267$), *Candidatus Saccharimonas* ($p<0.001$), *Lachnoclostridium* ($p=0.006$), *Muribaculum* ($p=0.020$), *Parasutterella* ($p=0.013$), *Rikenellaceae_RC9* ($p=0.044$), *Ruminococcus* ($p=0.006$) and *Tyzzerella* ($p=0.001$) and a decrease of the abundance of *Dubosiella* ($p=0.001$), *Eisenbergiella* ($p<0.001$), *Erysipelatoclostridium* ($p=0.001$), *GCA_900066575* ($p=0.052$), *Mucispirillum* ($p=0.006$) and *Ruminococcaceae_UCG_014* ($p=0.041$) were observed with age. Supplementations significantly affected the abundance of *Akkermansia* ($F_{(3,51)}=4.226$, $p=0.010$), *Alistipes* ($F_{(3,51)}=3.279$, $p=0.028$), *Bifidobacterium* ($F_{(3,51)}=3.319$, $p=0.027$), *Candidatus Saccharimonas* ($F_{(3,51)}=9.896$, $p<0.001$), *Clostridium_sensu_stricto_1* ($H_{(3)}=21.680$, $p<0.001$), *Faecalibaculum* ($H_{(3)}=14.300$, $p=0.003$), *GCA_900066575* ($H_{(3)}=11.000$, $p=0.012$), *Gemella* ($H_{(3)}=8.682$, $p=0.034$), *Ileibacterium* ($F_{(3,51)}=5.355$, $p=0.003$), *Lachnospiraceae_UGC_006* ($H_{(3)}=14.160$, $p=0.003$), *Muribaculum* ($H_{(3)}=8.459$, $p=0.037$), *Parabacteroides* ($H_{(3)}=16.770$, $p=0.001$), *Parasutterella* ($H_{(3)}=11.560$, $p=0.009$), *Rikenella* ($H_{(3)}=15.520$, $p=0.001$), *Romboutsia* ($H_{(3)}=18.710$, $p<0.001$), *Ruminococcaceae_UCG_014* ($H_{(3)}=17.520$, $p<0.001$), *Turicibacter* ($H_{(3)}=24.100$, $p<0.001$) and *Tyzzerella* ($H_{(3)}=26.540$, $p<0.001$). More precisely, PE supplementation increased the abundance of *Clostridium _sensu_stricto_1* ($p=0.006$), *Parabacteroides* ($p=0.002$) and *Turicibacter* ($p=0.008$), with a trend for *GCA_900066575* ($p=0.074$), *Lachnospiraceae_UGC_006* ($p=0.068$) and *Romboutsia* ($p=0.055$), and decreased the abundance of *Parasutterella* ($p=0.029$) and *Alistipes* (trend, $p=0.078$) compared to control. Om-3 supplementation induced an increase of the abundance of *Akkermansia* ($p=0.013$), *Candidatus Saccharimonas* ($p<0.001$), *Clostridium _sensu_stricto_1* ($p<0.001$), *Gemella* ($p=0.014$), *Lachnospiraceae_UGC_006* ($p=0.001$), *Parabacteroides* ($p=0.001$), *Rikenella* ($p=0.002$), *Romboutsia* ($p<0.001$) and *Ruminococcaceae_UCG_014* ($p<0.001$), and a decrease of those of *Bifidobacterium* ($p=0.011$), *Faecalibaculum* ($p=0.002$), *Ileibacterium* ($p=0.045$), *Muribaculum* ($p=0.050$), *Parasutterella* ($p=0.005$) and *Turicibacter* ($p=0.001$) compared to control. PE+om-3 decreased the abundance of *Muribaculum* (trend, $p=0.068$), *Parabacteroides* ($p=0.090$) and *Tyzzerella* ($p=0.003$) compared to control.
